# Improving occupational health for health workers in a pilot hospital by application of the HealthWISE international tool: An interview and observation study in China

**DOI:** 10.3389/fpubh.2022.1010059

**Published:** 2022-12-01

**Authors:** Min Zhang, Yiming Huang, Fengyao Wu, Dongmei Liu, Caiyun Wei, Yaqin Qin

**Affiliations:** ^1^School of Population Medicine and Public Health, Chinese Academy of Medical Sciences and Peking Union Medical College, Beijing, China; ^2^Nanning Fourth People's Hospital, Nanning, China

**Keywords:** occupational health, HealthWISE, health workers, interview, observation

## Abstract

**Objective:**

For a safe and healthy workplace in the health sector, the International Labor Organization (ILO) and the World Health Organization (WHO) jointly developed HealthWISE, an international technical tool that helps health workers (HWs) to identify workplace hazards and apply low-cost solutions. This study sought to gather experiences and lessons from a Chinese pilot hospital for the scale-up application of HealthWISE.

**Methods:**

A qualitative study was undertaken at a Chinese public hospital with a ≥5-year application of HealthWISE through in-depth interviews with targeted HWs who participated in the Training-of-Trainer (TOT) workshops, and observations were gathered using evidence from photos and publications, then, thematic analysis was formulated.

**Results:**

Driven by motivation, the participants learned from the HealthWISE TOT workshop alongside the favorite and worst parts of it. Positive changes and results of occupational health for HWs occurred after the workshop, the participants trained others and planned to implement HealthWISE within their responsibility. During the COVID-19 Pandemic, the Hospital acted the approaches of protecting the health, safety and well-being of HWs with significant results. Further suggestions on workshop and HealthWISE implementing as well as the national policies were collected. The study indicated the Hospital's experience of leadership and participation, supporting and facilitating, system establishment, and culture creation. The suggestion included keeping staff engaged under a positive safety and health culture, promoting recognition of HealthWISE among public health institutions nationwide, developing online courses for medical colleges, focusing on the alignment among various law systems, and adopting measures under the principle of the hierarchy of occupational hazards controls.

**Conclusion:**

This study has demonstrated the systematic improvement of occupational health for HWs by HealthWISE implementation in the Chinese hospital. The valuable experiences and lessons derived here can be shared with other hospitals in China and beyond, especially under the unprecedented challenges of the COVID-19 pandemic, to achieve the goals of safety, health, and well-being for HWs by building a resilient health system.

## Introduction

Health workers (HWs) are the main fundamental resources of the health system. The year 2021 was designated by the World Health Organization (WHO) as the International Year of Health and Care Workers under the theme “Protect. Invest. Together.” This announcement highlights the urgent need to invest in HWs to achieve shared dividends in health, jobs, economic opportunities, and equity by promoting decent working conditions, occupational health, and safety (1). Global research has indicated that investments in the health workforce and corresponding policy developments rely on good political leadership, coherent government strategies, institutional capacity, and intersectoral governance mechanisms (2).

In terms of occupational health, health-care institutions have been recognized globally to be among the most hazardous workplaces, carrying certain risks and hazards common to most sectors as well as others that are more specific to certain categories of HWs or to work practices in the health sector (1, 3). In general, HWs are exposed to a great variety of hazards in the workplace, which can be broadly divided into the following categories: biological hazards, chemical hazards, physical agents, poor ergonomic conditions, organizational problems, and psychosocial hazards (3).

As an extreme situation, the coronavirus disease 2019 (COVID-19) pandemic led HWs to face a variety of occupational hazards that put them at risk of disease, injury, and even death in the context of the COVID-19 response. In particular, these occupational risks included (a) occupational infections with severe acute respiratory syndrome coronavirus 2; (b) skin disorders and heat stress from prolonged use of personal protective equipment (PPE); (c) exposure to toxins because of increased use of disinfectants; (d) psychological distress; (e) chronic fatigue; and (f) stigma, discrimination, physical and psychological violence, and harassment (4).

As introduced in the HealthWISE Trainer's Guide and Action Manual, the International Labor Organization (ILO) works closely with the WHO to ensure work safety, health, and well-being in the health sector around the world. In 2010, the ILO and WHO jointly developed the technical tool, HealthWISE-Work Improvement in Health Services (hereafter referred to as HealthWISE), a practical, participatory methodology for improving the quality of health facilities, based on the principles of the ILO's Work Improvement in Small Enterprises program. In 2011, HealthWISE was piloted in a number of hospitals and health facilities in Senegal, the United Republic of Tanzania, and Thailand (4, 5). In 2012, HealthWISE was revised, then reviewed again by ILO and WHO specialists, and the tripartite expert working party completed an additional review in 2013 before its finalization. HealthWISE was official published in 2014, ILO and WHO jointly initiated the application of HealthWISE in international platform, however, there is no official information about how widespread is the use of HealthWISE over the world. HealthWISE is a combined action and learning tool consisting of two handbooks; of these, the Action Manual helps to initiate and sustain changes for improvement and is designed to promote learning-by-doing, while the Trainers' Guide contains guidance and tools for a training course. Basically, HealthWISE encourages managers and staff to work together to promote safe and healthy workplaces *via* the application of smart, simple, and low-cost solutions (4, 5).

Shortly after its publication, the HealthWISE application was advocated for as a sustainable national program in China by professional institutions. To facilitate its use in China, a series of actions have been undertaken to date, as follows. First, the Action Manual and Trainers' Guide were translated into Chinese, and their translations published in 2016, and the preface of the Chinese version was authored by the Vice Chairman of the Standing Committee of the National People's Congress (6–9). Second, a total of seven HealthWISE Training-for-Trainer (TOT) workshops were organized in China between November 2015 and August 2019, covering 130 hospitals and >450 health professionals across China (10–12). Third, the HealthWISE application was integrated into international and national occupational health policies, standards, and instruments—for instance, interventions on workplace violence and harassment prevention and control or the national standard Guideline for the Prevention and Control of Occupational Exposure to Bloodborne Pathogens (13–15). Fourth, with the implementation of HealthWISE, the China Sexually Transmitted Disease/Acquired Immunodeficiency Syndrome (AIDS) Prevention and Control Association established an Occupational Safety and Health Committee for HWs with the mission of improving the working conditions of doctors, nurses, technicians, and other workers in the health sector (16). Finally, the experiences of HealthWISE in China were shared globally by the ILO in the Sectoral Policies Department 2014–2015 Highlights; the Sectoral Policies Department 2016–2017 Highlights; the 2015 Labor Protection in a Transforming World of Work report; the Improving Safety and Health in Micro-, Small-, and Medium-sized Enterprises report; and the 2018–2019 United Nations AIDS Summary Report as well as in the international branch of the ILO human immunodeficiency virus (HIV) program in China and the 2020 Eliminating Poverty and HIV-related Employment Discrimination report (17–23).

To date, our program has published web information/articles on some aspects of the application of HealthWISE as mentioned above (6–23), and another pilot hospital in central China has also published an article on the primary needs of occupational hazards assessment and organizational improvement based on the HealthWISE checklist (24). Globally, we searched the literature with the key word “HealthWISE” since 2013 in the PubMed, only two literature were found related to the implementation of WHO/ILO HealthWISE, a study by Wilcox et al. in three African countries from 2016 to 2018 (25), and another study by Zungu et al. (26) in South Africa. Therefore, few reports of the global application of HealthWISE are available, and there are no studies about the effectiveness of HealthWISE as a whole in a Chinese health institution.

Under the unprecedented challenges placed by the COVID-19 pandemic on the promotion of occupational health of HWs, there is an especially urgent need for the international community to rapidly explore the implementation and promotion of HealthWISE by way of sharing experiences and valued lessons with each other. The purpose of this study was to verify the hypothesis whether the implementation of HealthWISE in a Chinese hospital provided systematic improvement for the occupational health protection among HWs.

## Methods

### Study design

This study used a qualitative study that combined interviews with observations, in order to collect experiences and lessons to support further expansion of the application of HealthWISE in health settings. The study focused on the ≥5-year application of HealthWISE in a Chinese public hospital, analyzing the opinions and evidence of organization-wide improvements in the occupational health of HWs. The interview focused on the personal perspective of HealthWISE application, while the observation study focused on the key photos and publications to demonstrate the substantial changes due to application of HealthWISE.

Under the context of the COVID-19 pandemic, ILO and Peking Union Medical College (PUMC) jointly initiated the rapid assessment program of the HealthWISE application in China in June 2020 for the purpose of sharing key findings of the assessment with stakeholders about recommendations to advance the occupational health of HWs.

Our proposal was approved by the ILO and PUMC, and the study reported in this article arose from one part of the rapid assessment, with the permission of the Hospital; thereafter, we carried out in-depth interviews and an observation study in September 2020 at the Hospital.

Our investigation team consisted of professionals from both PUMC and the Hospital, who were trained with respect to the background of the study and the basic concepts of HealthWISE. Team members were required to pay attention to the issue of reflexivity, focusing especially on interviewing the participants as equals to reduce the bias of the researcher and the research process during data collection and analysis.

### Characteristics of the HealthWISE TOT workshop

Both the Action Manual and the Trainers' Guide cover the HealthWISE topics in eight modules. Each module briefly introduces an issue and the objectives it aims to achieve, then sets out a checklist of four or five key points that are central to the topic and provides information and guidance on each point. Examples of good practice are also included.

Considering the legal conditions of occupational health of HWs in Chinese hospitals, four modules were prioritized in our HealthWISE TOT workshop—namely, controlling occupational hazards and improving workplace safety (module 1); musculoskeletal hazards and ergonomic solutions (module 2); biological hazards and infection control, with special reference to HIV and Tuberculosis (module 3); and tackling discrimination, harassment, and violence at the workplace (module 4).

Each of the previous seven HealthWISE TOT workshops in China held before the COVID-19 pandemic lasted, on average, 3 days. Speakers included members of Chinese national and local health authorities, members of the ILO headquarters and Beijing office, and leading experts on the program, and trainees were invited from local health authorities, trade unions, pilot hospitals, professional organizations, and non-governmental organizations to participate.

At each of the pilot hospitals, three representatives with key roles in the institution's function had one opportunity of participating in HealthWISE TOT workshop, including the hospital director, the departmental director, and frontline health professionals. In addition to the workshop, our national program provided follow-up technical support to the pilot hospitals, including field visits and guidance, remote discussion, and summaries for improvement. Between the workshops, learning-by-doing was promoted, and continual corrective actions were taken by the pilot hospitals using a step-by-step approach. By means of active learning, two-way communications were highlighted; any progress made and lessons learned after the previous workshop were shared with the participants of the next HealthWISE TOT workshop and other communication events.

### Study setting

This study was carried out at a provincial hospital in southern China, which is a tertiary hospital with 550 beds and 850 staff (hereafter referred to as “the Hospital”). The Hospital specializes in infectious diseases and comorbidities, particularly, HIV/AIDS, Tuberculosis, and Hepatitis. It has been the hospital designated to treat COVID-19 patients in the region of the provincial capital city since January 2020.

The Hospital has been actively engaged in the application of HealthWISE since 2015. Staff of the Hospital participated in five of the HealthWISE TOT workshops, with the motivation of representing one of the pilot hospitals in the initiative.

### Interview data collection

Before the interviews commenced, a semi-structured questionnaire was drafted in both English version and Chinese version which was validated by ILO officers and our lead team member. The finalized questionnaire focused on four themes with 14 questions (Table 1), the Chinese questionnaire was applied in the interview.

**Table 1:** Interview questions about the application of HealthWISE.

**Theme**	**No**.	**Question**
Learning from the workshop	1	When did you attend HealthWISE TOT Workshop?
	2	Why did you attend the HealthWISE TOT Workshop?
	3	What did you learn from the course?
	4	What were the top-two favorite parts of the course?
	5	What was the worst part of the course?
Actions after the workshop	6	What has happened to the individual, team or hospital after receiving HealthWISE TOT Workshop?
	7	Have you trained other HWs after receiving HealthWISE TOT Workshop? If so, how many? If not, why not?
	8	What are the results of the implementation of HealthWISE in hospital? Can you give some examples?
	9	Based on your job responsibilities, what plan are you going to carry out to implement HealthWISE at work?
Practice during COVID-19	10	What do you think the approach to protecting the health, safety and well-being of HWs during COVID-19?
	11	Did your hospital implement/enhance HealthWISE during COVID-19? What was the result?
Further suggestion	12	How can HealthWISE TOT Workshop be improved? How to enhance the implementation of HealthWISE in your hospital?
	13	In your opinion, what are the bottleneck problem that should be addressed when establishing the occupational health protection system for HWs at the national level?
	14	Before the interview is over, do you have anything else to tell us?

Under the coordination of the managerial department of the Hospital, HWs in the Hospital were selected as purposive interviewees. Participants included (a) HWs who had participated in the HealthWISE TOT workshop or trained by the HealthWISE TOT workshop trainer, (b) HWs who had promoted good practices for occupational health in the Hospital, (c) HWs who are on the position of the Hospital director, departmental director, Trade Union director, frontline doctor, frontline nurse, and (d) HWs who voluntarily provided verbal informed consent for enrollment.

As a result, we interviewed ten HWs. More specifically, of the study participants, five had participated once in a HealthWISE TOT workshop, three had participated twice, and one had participated three times, while the last participant had read the HealthWISE Action Manual under the guidance of trainers of the HealthWISE TOT workshop. Two participants were hospital directors, four were departmental directors, one was frontline doctors, two were frontline nurses, and one was a professional of occupational health management. Seven participants were female and three were male.

Due to the restriction measures put in place in response to the COVID-19 pandemic, personal interviews were conducted remotely *via* telephone or the WeChat social media platform (Tencent Holdings Limited, Shenzhen, China), with each interview lasting about 30 min in Chinese. At the start of the interview, the interviewees were informed about the study background, research purpose, and definitions of relevant terms. Then, after the interview, records of the 10 interviewees were filed anonymously under numbers P1–10 to preserve the privacy of the interviewees.

For the purpose of objective analysis, individual in-depth interview was interlinked with observation study in particular three parts: (a) Question 6 and 8 in the questionnaire which covered What happened and Results after TOT; (b) Question 11 in the questionnaire which covered Results of Practice during COVID-19; and (c) Question 12 in the questionnaire which covered the Further Suggestions. After sharing their opinion of the question, the interviewee had been invited to provide information about evidence of actions and changes, then, the evidence will be included into the data collection of observation study.

### Observation data collection

“Change” is the typical feature and practical essence of HealthWISE, the word “change” appears more than 70 times in the HealthWISE Action Manual (7), for instance, “sustaining changes for improvement,” “the fullest sense of ownership of the change process.” There are six steps in Planned approach to sustainable improvements, the sixth step is “Monitor, review and keep improving,” take picture is a powerful tool to demonstrate the achievements.

As mentioned above, the observation study aimed to collect objective evidence of organization-wide actions and improvements in the occupational health of HWs, it played the complementary role with the in-depth interview, especially the question 6, 8, 11, and 12 in the questionnaire.

According to the approach of HealthWISE, implementing of HealthWISE in the Hospital is led by manager-worker collaboration and applies core WISE principles: (a) Build on local practice and resources; (b) Focus on achievements; (c) Promote learning-by-doing; and (d) Encourage exchange (5). Therefore, the key of HealthWISE application were actions and changes which had been summarized as photos and publications of the Hospital. During the data collection period, using the information of the interviewees, our investigating team member observed all evidence on the effective application of HealthWISE in the Hospital; on the one hand, they identified changes in practices, equipment and the environment by more than 30 photos on-site; on the other, they collected eight publications authored by the staff of the Hospital relevant to occupational health.

### Interview data coding and analysis

All interview recordings were transcribed verbatim in Chinese, the interviewees' characteristics (job title and interviewee number from P1 to P10) were mentioned to keep the quotes anonymous, instead of their names. Thematic analyses of the interview data in Chinese were undertaken using Microsoft Excel (Microsoft Corporation, Redmond, WA, USA) and NVivo version 12.0 (QSR International, Doncaster, Australia), the six-phases thematic analysis suggested by Braun and Clarke (27, 28) was conducted mainly by the corresponding author (MZ) and the second author (YH): (a) Familiarization with the data. Independently, the two authors repeatedly read the whole transcript in order to become intimately familiar with the data. (b) Generating initial codes. The themes were based on the themes in the semi-structure questionnaire, the initial code of subthemes was drafted by MZ and YH independently. (c) Search for the themes. The coded data is reviewed and compared with the different initial subtheme from MZ and YH. (d) Review the themes. Two external members outside the investigation team were invited to participate in the iteration process of subthemes and codes with the two authors. (e) Defining and naming the themes. From the professional perspectives of HealthWISE implementation, the codebook was finally formulated (Table 2). And (f) Write a research report. The meanings behind the transcribed text were analyzed by the subthemes and codes in Chinese, then the report was translated into English.

**Table 2:** Codebook for interview analysis among participants of HealthWISE TOT workshop.

**Themes**	**Categories/subthemes**	**Codes**	**Data sources**
1. Learning from the workshop	1.2 Motivation	1.2.1 External expert guidance 1.2.2 Curiosity driving 1.2.3 Job improving	Interview question 2
	1.3 Gains	1.3.1 Awareness raising 1.3.2 Knowledge gaps filling 1.3.3 Work practice formalization 1.3.4 Targeted oriented	Interview question 3
	1.4 Two favorite parts	1.4.1 Training module 1.4.2 Participatory approach 1.4.3 Participant empowerment	Interview question 4
	1.5 The worst part	1.5.1 Not mentioned 1.5.2 Worst part mentioned	Interview question 5
2.Actions after the workshop	2.1 What happened	2.1.1 Establishing OSHM System 2.1.2 Improving Workplace	Interview question 6
	2.2 Train others	2.2.1 Internal training 2.2.2 External training	Interview question 7
	2.3 Results	2.3.1 Enhancing solidarity and engagement 2.3.2 Reshaping mindset 2.3.3 Repeating the aforementioned changes	Interview question 8
	2.4 Individual plan	2.4.1 Implementing IPC 2.4.2 Continuing focus on training 2.4.3 Sustainable engagement in OSHM System 2.4.4 Continuing application of HealthWISE	Interview question 9
3. Practice during COVID-19	3.1 Approaches	3.1.1 Good work organization 3.1.2 Personal protection 3.1.3 Social support	Interview question 10
	3.2 Results	3.2.1 Treating patients well 3.2.2 Eliminating fear and anxiety 3.2.3 Standardized personal protection	Interview question 11
4. Further suggestion	4.1 Suggestion on HealthWISE	4.1.1 Improving TOT 4.1.2 Improving Implementation	Interview question 12
	4.2 Bottleneck problem	4.2.1 Specific legislation 4.2.2 Earmarked budget 4.2.3 High-quality trainer	Interview question 13

### Observation data analysis

Based on the collection of more than 30 photos on-site and eight publications, MZ and YH discussed the professional value of changes from those evidence, firstly, they selected the pictures to indicate the typical measures of HealthWISE application, secondly, they selected the descriptions in publications to demonstrate the improvement of HealthWISE application.

### Patient and public involvement

No patient or member of the public was involved in the design, recruitment, or conduct of this study. HWs in the Hospital participated in the study. The findings of this study were disseminated to the Hospital in September 2021.

### Ethics

This study was automatically approved on the ethical review by PUMC through approval of the ILO and PUMC program of rapid assessment of the HealthWISE application in China according to internal regulations. All personal identifiers (name, contact information) of participants were removed from the dataset before analysis.

## Results

### Learning from the HealthWISE TOT workshop

The opinions from interviewees about learning during the workshop were summarized under four subthemes: motivation for participation, gains from the workshop, top two favorite parts of the workshop, and the worst part of the workshop.

First, the motivation of the participants of Health TOT workshop was analyzed by the following codes: (a) External expert guidance. The training was recommended by a senior expert from the provincial professional association (4/10 participants); (b) Curiosity driving. It was the first time to know the issue of occupational protection for HWs (5/10 participants); and (c) Job improving. The responsibilities of departmental managers, such as nursing, infection control, frontline doctors, required them to update knowledge to improve their competencies (4/10 participants).

The motivation was particularly expressed in the following interviewee quote:

“*… the vice president of Guangxi Provincial Association of STD/AIDS Prevention and Control recommended us to participate in the TOT workshop on occupational protection for HWs. …I usually have the idea that occupational protection is only related to workers or miners who were exposed to occupational dust.” (vice director of the Hospital, P1)*

“*Since my position now is in the Infection Prevention and Control Department, and a big part of my job is dealing with occupational exposures in the hospital, I wanted to see if there was any new progress in the reporting and recording measures of occupational exposures.” (frontline physician of Infection Prevention and Control Department, P5)*

Second, the following four codes covered the gains from the workshop: (a) Awareness raising. The participants recognized that occupational health protection for HWs was a comprehensive protection (9/10 participants); (b) Knowledge gaps filling. The participants learned the comprehensive knowledge of HWs occupational protection in a systematic way (8/10 participants); (c) Work practice formalization. The participants improved the procedures and habits of usual practices (2/10 participants); and (d) Target oriented. The participants understood how to act toward the right direction of occupational protection for HWs in the future (2/10 participants).

The gains were specifically reflected in the following interviewee quote:

“*The first was the mindset changing through the TOT workshop, I had a broader understanding of occupational protection. In fact, occupational health protection for HWs was comprehensive for both physical and psychological health. It was beneficial to the safeguard of workers' rights, and the sustainable development of the whole health sector.” (vice director of the Hospital, P1)*

“*…it taught me the knowledge of occupational safety and protection in a very systematic way, such as biological and physical hazards, environmental health, ergonomics, anti-discrimination and so on, while [the previous training] was fragmented.” (director of the Nursing Management Department, P3)*

“*I think what we learned is quite practical, the TOT workshop analyzed some details that we usually ignored in our work. And also, we HWs did not pay much attention to the prevention of some potential occupational diseases arising out of exposure during medical practice.” (frontline nurse of the Blood Purification Department, P8)*

“*I didn't know much about HealthWISE before,... now I know where I'm going forward.” (occupational health professional of the Occupational Health Protection Department, P10)*

Third, the two favorite parts of the TOT workshop were analyzed by three codes: (a) Training module. Two participants expressed that they liked all modules, and another two participants who were responsible for infection prevention control expressed their preference for the biological hazards and infection prevention and control modules (4/10 participants); (b) Participatory approach. The participants favored the action-oriented participatory approach, including group discussions, peer interaction, walk-through survey onsite, presentations based on work in a group, and so on (5/10 participants); and (c) Participant empowerment. The TOT workshop empowered participants to use HealthWISE to identify problems at the worksite and propose practical solutions (5/10 participants).

“*HealthWISE training programme was different from others, it focused on the interactive process between facilitators and participants, while many other workshops only used the traditional way of “trainers talking and students listening.” Another exciting part was that the workshop organized us to visit several units of the hospital to identify good practice and problems, after that, we made PPT presentations together to report our findings...” (frontline physician of Infection Prevention and Control Department, P5)*

“*I was impressed by the group discussion, we could express our own points, then we discussed and summarized our findings and solutions, we made a brief PPT together and chose one participant to report on behalf of our group. This experience was helpful for us to improve our ability of giving speech. Because I used to rush and be tired very much, and had less time to participate in such activities in my routine work, so I felt quite good to participate in [this kind of TOT workshop].” (frontline nurse of the Blood Purification Department, P8)*

Finally, six participants expressed that there was no worst part in the workshop, while four participants addressed some weaknesses as follows:

“*The training period is short, the content of the training is a little less or is not in-depth enough, maybe because of the limitation of our own professional knowledge...” (vice director of the hospital, P1)*

“*I think it may be my own problem, I am not so good at applying ergonomics (to practice) very well. Even though I trained ergonomics [to other HWs], I thought the promotion in this area was not so good, the ergonomics changes in the real world is not only depended on my personal efforts, but also depended on [the efforts of] the whole hospital and the awareness of staff... Additionally, the awareness of anti-discrimination should be raised, the action to anti-discrimination should be focused.” (director of the Nursing Management Department, P3)*

“*I did not feel so good about the training period, it seemed a little bit long...the training period may actually be shortened.” (frontline physician of Infection Prevention and Control Department, P5)*

“*The main problem was that the motivation of some participants may need to be inspired.” (occupational health professional of the Occupational Health Protection Department, P10)*

### Actions after the HealthWISE TOT workshop

The action after the HealthWISE TOT workshop from interviewees and observation were summarized under four subthemes: changes of improvement, training others, results and individual plan.

First, the changes for improvement happen in the Hospital were analyzed by two codes: (a) Establishing Occupational Health and Safety Management (OSHM) System (3/10 participants), in particular: (1) The Hospital established the Steering Committee of Occupational Safety and Health in 2016, whose members consisted of leaders of the Hospital and relevant departments. The Department of Occupational Health was officially developed in 2019 with the aim to protect the occupational health of HWs in accordance with the Law on the Prevention and Control of Occupational Diseases; (2) The standard operation procedure (SOP) was established for occupational protection during service delivering for patients with infectious diseases like HIV/AIDS, Tuberculosis and Hepatitis; (3) Adopting protective measures for key units such as negative-pressure wards and fever clinics; (4) Enhancing training for all HWs, including temporary workers (such as cleaners); (5) Setting up inspecting groups, carrying out daily supervisions and inspections; (6) Personal protection was enhanced in a systematic way, such as improving HWs' well-being, providing social psychological support and counseling, ensuring the PPE quality and sufficient supply.

(b) Improving workplace for safety and health (7/10 participants), including: (1) reducing extra beds at corridors in the inpatient units; (2) improving operative equipment, adjusted workstations for reducing bending operations at work, modified poor ergonomic conditions with an investment of 1.5 million RMB¥ (about 214,000 US$); (3) Adopting specific quarantine measures to avoid patients with different infectious diseases visiting each other; and (4) Enhancing security measures with increasing security staff and installing alarming devices.

**Figure 1:**
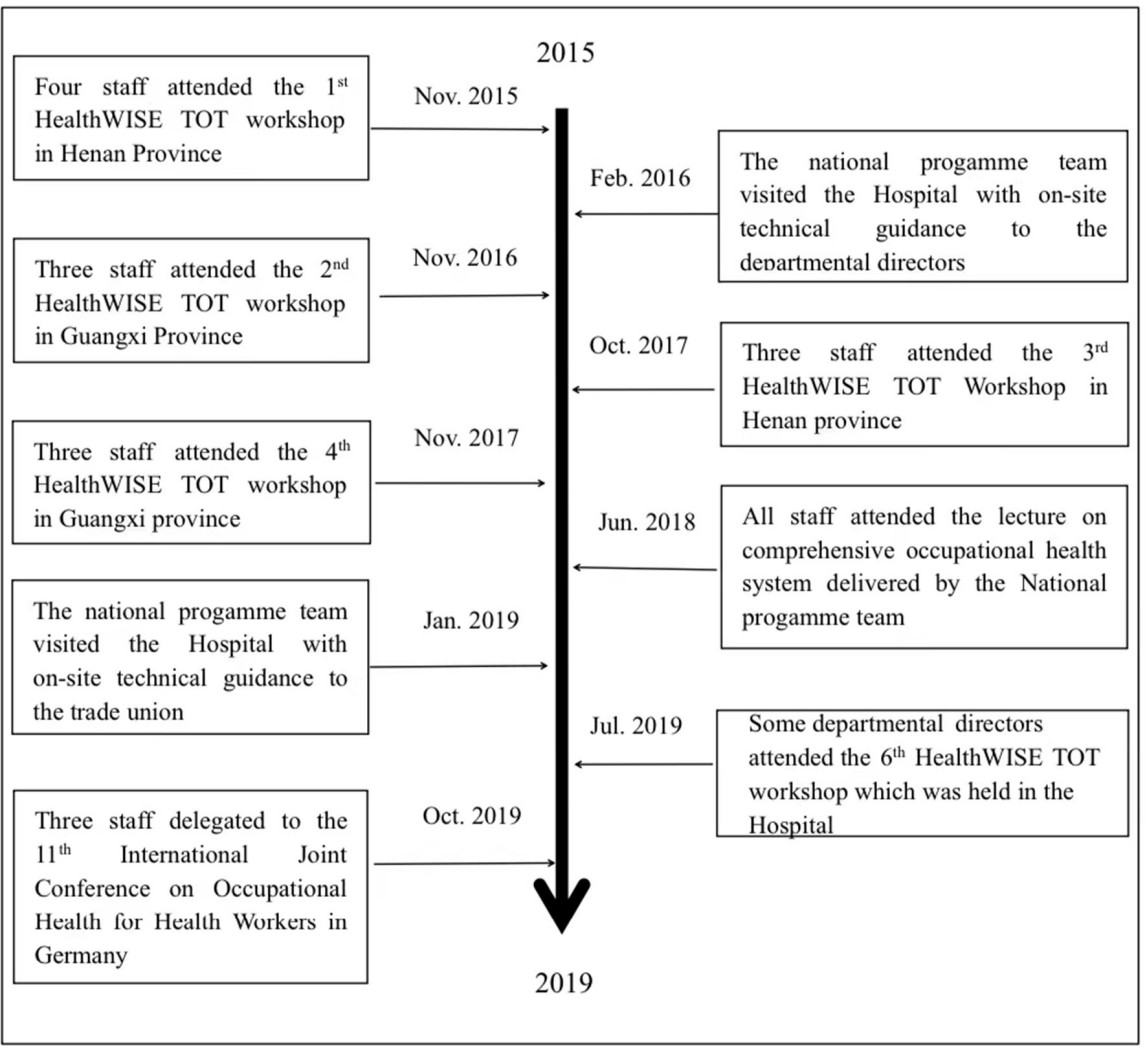
The milestones of external technical guidance. in the hospital from 2015 to 2019.

**Figure 2:**
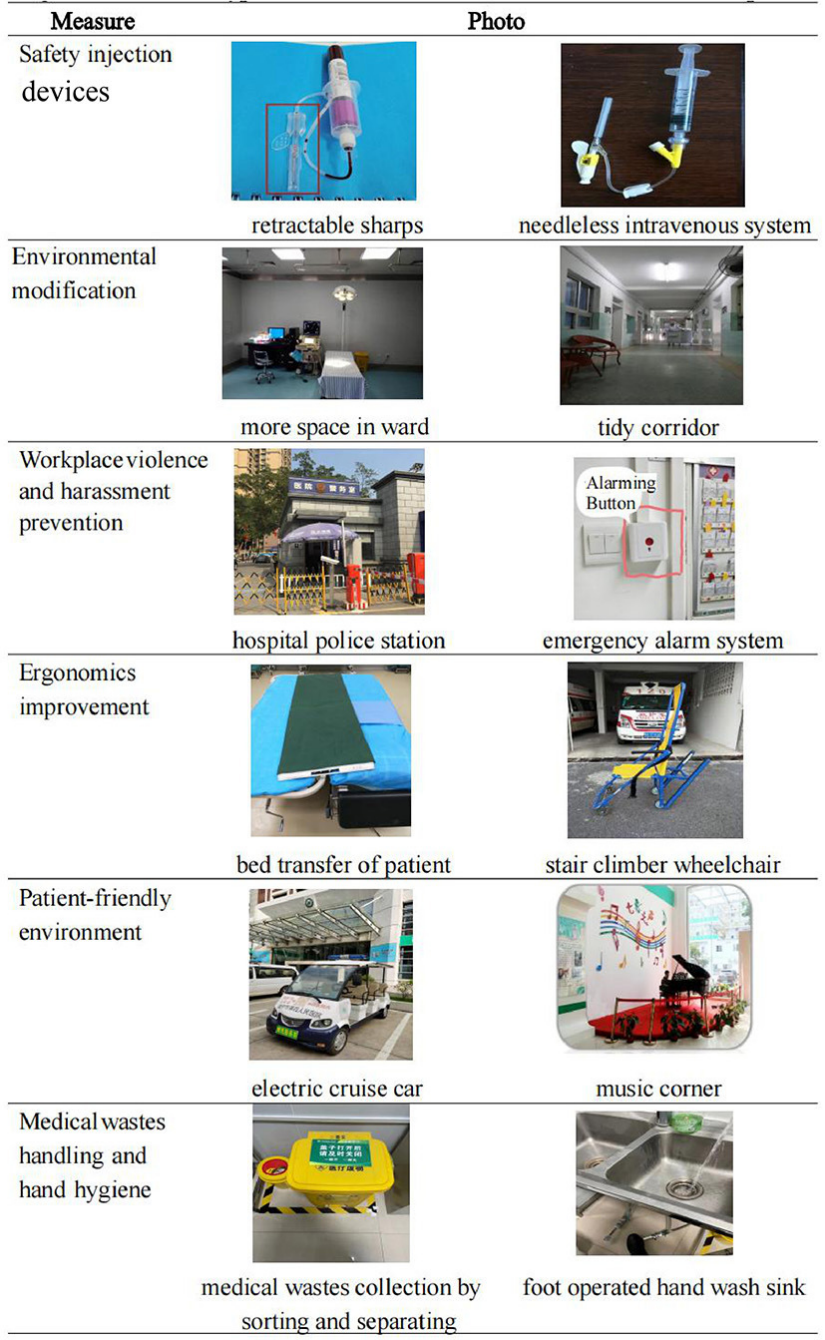
Photos of typical measures based on the HealthWISE in the hospital.

As a result of observation study, the milestones of external technical guidance from 2015 to 2019 were identified in Figure 1, the photographic evidence of the typical measures based on HealthWISE implementation was shown in Figure 2.

Figure 1 marked four kinds of milestones in HealthWISE training, including five HealthWISE TOT workshops, one international conference of occupational health for HWs, the lecture on occupational health for HWs provided by national programme team, and two runs of on-the-spot guidance from the national programme team.

In Figure 2, the photos covered six measures of occupational health for HWs: (a) In the safety injection devices measure, retractable sharps and needless intravenous system were applied for all services in the Hospital. (b) From perspective of environmental modification, more space in ward was available as a result of improving operative equipment and workstations, and tidy corridor were achieved due to reducing extra beds at corridors in the inpatient units. (c) In order to prevent workplace violence and harassment, a hospital police station was established, emergency alarm system was adopted. (d) During ergonomics improvement, bed transfer of patient and stair climber wheelchair were adopted for prevent musculoskeletal disorders among HWs. (e) For the patient-friendly environment, electric cruise car was used for patients free-of-charge, light music was played at music corner in the outpatient hall. (f) In terms of medical wastes handling and hand hygiene, medical wastes collection was sorted and separated from non-medical wastes, foot operated hand wash sinks were installed in many workplaces and public places for both HWs and patients.

The improvements were particularly reflected in the following interviewee quotes:

“*[In the past], hospital beds were not sufficient for patients, so we added as many beds as we could in the corridors at the inpatient units. After the TOT training, in this regard, we changed our mind, we needed to improve our environment, ventilation and corridors with more space to ensure the health and safety of the staff...now we do not add extra beds in the corridors at the TB inpatient wards, it is a big change in the hospital.” (director of the Nursing Management Department, P3)*

“*[Before the HealthWISE application], the steel wheel of the nurse's trolley made a noise and, at night or midnight, nurses were reluctant to push it into the ward with injections for patients; instead, they used to take a small tray for operating without a sharps disposal container, making it is easy for a sharp injury to occur. Now, we equip with a smaller plastic trolley with little noise, and the nurses like to stock it with necessary equipment, including a sharps disposal container. Thus, occupational hazards exposure is avoided.” (frontline physician of Infection Prevention and Control Department, P5)*

“*We are working at the blood purification department...After the TOT training, we discussed with the director of nurse about the problems existing in the practice and what kind of occupational diseases would potentially arise out of our routine activities. Later, we did some improvements, such as equipment of a workstation for the nurses' operation, so we did not need to bend low back too much at work; it was so crowded before...now the work environment is very comfortable, I feel very happy.” (frontline nurse of the Blood Purification Department, P8)*

“*After the application of HealthWISE, the significant improvements in our hospital are that the protective materials are supplied sufficiently, the supervision is strengthened, and someone makes sure the prevention strategies and PPE meet the requirements.” (occupational health professional of the Occupational Health Protection Department, P10)*

Second, after receiving the training of HealthWISE TOT workshop, ten interviewees made contribution to a series of on-the-job trainings as the trainer/lecturer in person or online, their roles was analyzed by two codes: (a) Internal training. HealthWISE training courses were integrated into daily training for all staff, pre-service training for new HWs, special training for IPC (Infection Prevention and Control), occupational safety and health (OSH), and disease protection training (5/10 participants); (b) External training. HealthWISE training courses were tailored to provincial trainers outside the Hospital, which were completed through various continuous education programs, academic conferences, and training courses for medical students in nursing schools (5/10).

The trainer's particular efforts were reflected in the following interviewee quotes:

“*I not only shared some experiences of our routine work at last year's training, but also introduced these ideas to other HWs in the workplace...” (vice director of the Hospital, 1)*

“*[Our hospital] has training program, every year before new HWs start their work, they need to be trained at first. At the same time, the hospital organized some training workshops every year, I also prepared some materials to train my colleagues, …training workshops were at least two or three times a year, and 150-200 participants participate every year.” (director of the Nursing Management Department, P3)*

“*As a member of Hospital Quality Control Center at Nanning City, I also trained HWs on hospital IPC of COVID-19 from other medical institutions and grassroots units in Nanning City. The contents were accessible on the MOOC app, all HWs in Nanning City can learn it online.” (director of the Infection Prevention and Control Department, P9)*

Third, as a result of HealthWISE application in the Hospital, the improved knowledge and attitude among HWs was analyzed by three codes: (a) Enhancing solidarity and engagement. HWs started to actively safeguard their rights and interests of occupational health, teamwork had been strengthened, the Hospital entered the trajectory of sound development (2/10 participants); (b) Reshaping mindset. The mindset of HWs was shifted from refusing changes to accepting changes, furthermore, took proactive measures, the feeling of HWs at work was changed from fear to subtle fear, then achieved better mental health (3/10 participants); and (c) Repeating the aforementioned changes for improvement happen in the Hospital (5/10 participants).

The particular results were reflected in the following interviewee quotes:

“*Also, we have a new mindset after the TOT. First of all, the daily training and departmental management will share the information of rights of HWs, ensure they keep the concept at work. The team can develop positively only when everyone is well. So if we did a good job on occupational health, including (the protection of) emerging infectious diseases, there would be less adverse effects in the future, the whole team would develop in an active and positive way.” (vice director of the Hospital, P1)*

“*As a result [of applying HealthWISE], in particular, the staff's awareness of occupational exposure, mental health and occupational injuries are raised, they are able to accept the fact [of occupational hazards], and adjust work style accordingly in some degree. Moreover, the orientation of the whole system [of occupational health] has been improved. The work processes need to be changed, like PPE, working hours, fatigue situation, off-peak service, especially, the coordination and operation worked well for urgent surgical operations during COVID-19.” (director of the third ward of Infectious Diseases Department, P4)*

“*The trained staff are less fearful about the job for infectious diseases. They feel it is easier to perform their duties, not like the situation was when they started to work in the department of HIV/AIDS or Tuberculosis. It is particularly significant among my colleagues…HWs working at TB and HIV/AIDS departments used to wear medical masks, now they are always provided with N95 respirators.” (director of nurse in AIDS Outpatient Department, P6)*

In terms of family-friendly measures by our observation study, the Hospital developed the policy of maternity leave for female employees; besides the national regulation of 6–7 months of legal maternity leave, “a female employee can continue to take extra months of maternity leave until her baby grows up to >1 year old.” During the summertime, the trade union invested 200,000 RMB¥ (about 28,600 US$) annually to organize a summer camp within the Hospital for employees' children on summer vacation so that their parents could keep working routinely at the Hospital without worrying about childcare; notably, this initiative has ultimately benefited >100 employees and their families.

Prevention and control of occupational exposure to bloodborne pathogens was prioritized in the Hospital, especially when withdrawing and sealing indwelling needles, injecting drugs or drawing blood, or disposing of medical waste (29). The internal report showed that notifications of occupational exposure in the Hospital dropped persistently over time; for instance, for each year from 2017 to 2020, occupational exposure episodes to bloodborne pathogens totaled 33 cases, 21 cases, 20 cases, and 10 cases, respectively. No infection was confirmed among these cases. According to a study by the Hospital in 2016, the acknowledgment rates among HWs were 94.69% (HIV occupational exposure), 92.31% (wound-treatment measures after occupational exposure), and 92.63% (antibody-detection time after occupational exposure), respectively. Preventive measures for HIV/AIDS occupational exposure were implemented, including privacy protection, post-exposure prophylaxis preparedness and implementation, mental health support, counseling services, regular training, financial support, and so on (30).

Fourth, the interviewees' plans to implement HealthWISE at work based on their job responsibilities were analyzed by four codes: (a) Sustainable engagement in OSHM system. This includes promoting the mechanisms of cooperation and coordination within the hospital, strengthening the competency-based occupational health expertise (2/10 participants); (b) Continuing focus on training. The main points in communication will include daily guidance during service delivering at the department level, pre-service training for new HWs (3/10 participants); (c) Continuing application of HealthWISE (2/10 participants); and (d) Implementing IPC, improving bedside hand hygiene, completing the three doses of the HBV vaccine for new recruiting HWs (3/10 participants).

The particular individual plans were reflected in the following interviewee quotes:

“*...Some of the new staff were tested negative antibody of Hepatitis B, we suggested the hospital leaders to provide three doses of the HBV vaccine for these colleagues, and conduct a follow-up test to ensure that they have antibodies...” (director of the Nursing Management Department, P3)*

“*Besides, the hospital should supply sufficient medical protective equipment, for example, the hospital must provide safe needles and containers for sharp instruments when drawing blood, and needleless system in the wards in the infection department and outpatient clinic.” (director of nurse in AIDS Outpatient Department, 6)*

“*We are constantly improving patient management...The blood purification department is in a hall with centralized patient management, thus we need to improve hand hygiene [during the practice] among beds to prevent cross-contamination. HWs have a lot of chances to expose to blood during operation, so we need to enhance occupational prevention. I will start from these points.” (frontline nurse of the Blood Purification Department, P8)*

“*In terms of the administrative responsibility, the best we can do is to train HWs and enhance the supervision in our routine. We hope that through enhanced training, our staff can realize that enjoying occupational safety is also one part of their rights and interests, and they can raise their awareness of (occupational hazards) prevention and control.” (director of the Infection Prevention and Control Department, P9)*

### Practice during the COVID-19 pandemic

After the COVID-19 outbreak began in the early of January 2020 in China, the Hospital became the designated hospital for COVID-19 treatment within its region, a solid basis was set by years of application of HealthWISE for the fight against COVID-19. The Hospital adopted comprehensive and rigorous measures against multiple occupational hazards which were summarized as two subthemes: the approaches of protecting the health, safety and well-being of HWs as well as its results.

First, the approaches were analyzed by three codes: (a) Good work organization (4/10 participants). (1) With leadership commitment, human and other resources were allocated to support HWs with high-quality PPE, the comprehensive protective measures were in place; (2) The Hospital shortened the working hours of fonrtline HWs as <8 per day with mandatory shift work, ensured they take 14 days' leave for every 15 working days; (3) The Hospital implemented emergency response measures, provided training of IPC, occupational protection and mental health.

(b) Personal protection. The Hospital stocked qualified and sufficient PPE, gave top priority to frontline HWs, made sure that every HW is trained to use the PPE correctly, those measures covered temporary workers, such as cleaners and security workers (3/10 participants).

(c) Social support. Based on the thematic survey on psychological status of HWs, the Hospital provided psychological counseling, traditional Chinese medicine for enhancing physical quality, salary subsidies, logistics support and tailored interventions (3/10 participants).

The particular actions were reflected in the following interviewee quotes:

“*Five medical workers were dispatched to the Hubei (the hardest-hit area) medical team for epidemic control. We trained them on occupational safety and health protection before the mission; the hospital leaders communicated to them with inspiration and showed leadership commitment to fully support them.” (director of the Hospital, P2)*

“*All staff in our hospital were trained, including health professionals and supporting staff (such as cleaners and security guards). When this study was conducted, we had achieved the goal of zero infections of COVID-19 among HWs.” (director in the Occupational Health Protection Department, P9)*

“*At that time, our hospital provided well-organized logistical support for the frontline HWs. A temporary subsidy was paid... frontline staff were offered 14 days off for every 15 working days to reduce their work stress.” (occupational health professional of the Occupational Health Protection Department, P10)*

Second, results of protecting the health, safety and well-being of HWs were analyzed by three codes: (a) Treating patients well. The quality of medical care was controlled with reduced medical malpractice, the service to COVID-19 patients were improved with “zero deaths of COVID-19 patients” (3/10 participants); (b) Eliminating fear and anxiety. Sound work-rest balance and support system were developed to reduce HWs' stress and fatigue (3/10 participants); and (c) Standardized personal protection. Every HW had the know-how of applying standardized personal protection for their own safety with “zero infections of HWs”.

The particular results were reflected in the following interviewee quotes:

“*...These were specific examples of the practice for the [occupational] protection system, which reflected our care and support for HWs from the material supply to humanistic care. [In this way,] the HWs were happy at work, and the occurrence of malpractice and mental illness could be reduced.” (vice director of the Hospital, P1)*

“*At the beginning of COVID-19 outbreak, there was [PPE] shortage in the hospital, and each of us felt a little bit panic, but later with the fully support from all parties, we went through with safe...” (director of nurse in AIDS Outpatient Department, P6)*

It was demonstrated in observation study that, after years of fostering, the core value of “Guard for Lives” was formulated, and the hospital culture has been a strong engine for improving the core competitiveness, which led to the sustainable development of occupational health. Meanwhile, cultural advantages were demonstrated in the fight against COVID-19 (31, 32). From January to March 2020, >2,000 patients with fever were admitted to the Hospital, including both suspected and confirmed COVID-19 cases; eventually, however, all cases were cured and discharged. The adopted scientific, standardized measures laid a solid foundation for the effectiveness of prevention and control, clinical diagnosis, treatment, and nursing management, protecting patients and HWs alike (33, 34).

### Further suggestions

The further suggestions were focused on two subthemes: improving the HealthWISE TOT workshop and the implementation of HealthWISE in the Hospital, and the bottleneck problem of occupational health protection system for HWs at the national level.

First, participants indicated their suggestions on HealthWISE by two codes: (a) Improving TOT (7/10 participants). (1) TOT enlarging. The TOT workshop should be integrated into the education for medical students in medical schools, orientation for new HWs in health care institutions. The participants of the TOT workshop should be extended from HWs at IPC and nursing units to all HWs; (2) Training approach innovation. By making digital videos, the TOT workshop could be conducted as long-distance training and self-guided learning online; (3) Training materials modification. The training materials should be tailored with clinical practice and cases, in order to improve the feasibility.

(b) Improving implementation. Total involvement, including all department and staff engagement in the Hospital, is the key for implementation of HealthWISE (3/10 participants).

The particular comments were reflected in the following interviewee quotes:

“*HealthWISE training was quite good, the discussion session was somewhat like a revolving class...” (frontline nurse of the Infectious Diseases Department, P7)*

“*…I thought the content of basic theory was very comprehensive, it was well-prepared, it would be more effective if the medical cases were combined to explain and analyze [the protective measures] during the training…” (director of the Nursing Management Department, P3)*

“*Nowadays, because of the impact of COVID-19, adopting an online training model would be better for practice.” (director of nurse in AIDS Outpatient Department, P6)*

“*Promoting the application in the hospital could not get the effectiveness in a short time.... I would like to consider the issue [of occupational health] combining the work practice of IPC and other health care units, sharing good experiences, especially what we learned in COVID-19. [Frontline HWs in] different units needed to get more support from the hospital leaders for this, which was very meaningful.” (director of the Nursing Management Department, P3)*

Second, when establishing the occupational health protection system for HWs at the national level, the bottleneck problems which should be addressed were analyzed by three codes: (a) Specific legislation. The national legislation should focus on the issues related to occupational protection for HWs, including authorization of the responsibility and function to competent authorities, specifying the occupational health rights and interests of HWs, capacity building, annual paid leave, coordinated mechanism of diagnosis/recognition, reporting/recording/notification and compensation for occupational HIV/AIDS, implement occupational health supervision (6/10 participants); (b) Earmarked budget. Establish a national budget for PPE and occupational protection in health care facilities (2/10 participants); and (c) High-quality trainer. The national expert network should be enlarged for the large-scale of training (1/10 participants).

Specific comments were offered by the interviewees, as follows:

“*It should authorize competent authority for occupational health protection among HWs by the specific national law, otherwise, we don't know where to notify.…There should be expert in the competent authority, or part-time experts, our country needs to invest in this area. …investment is also necessary in training and public communication.” (vice director of the Hospital, P1)*

“*...Actually, many hospitals have funding problems for occupational protective equipment, I hope the country arranges some specific funding for protective equipment for HWs. For example, during the COVID-19, N95 respirators were seriously shortage, therefore they were very expensive at the beginning of the COVID-19 outbreak, which was a very heavy burden for the hospital and its units.” (director of the Infection Prevention and Control Department, P9)*

The observation study has gathered the recommendations about the long-term mechanism in the Hospital: (a) optimize the work practice and flow; (b) raise the awareness of occupational health and safety of HWs; (c) standardize the utilization of protective equipment; (d) strengthen hospital supervision; (e) avoid both insufficient and overprotection; (f) maintain law-based scientific prevention and control measures; (g) minimize the incidence of occupational exposure and avoid nosocomial infections, (h) maintain a “wartime mode” to battle an outbreak of COVID-19; (i) emphasize previewing of triage, fever clinic, and isolation wards; (j) strictly supervise the entrance, care, and exit of patients at the hospital; and (k) tackle the barriers of talent attraction and retention (35–37).

## Discussion

### The theoretical features of rapid assessment with learning-by-doing

HealthWISE is a combination of action and training that encourages leadership and health care professionals to work together for the health, safety, and well-being of HWs in the workplace, helping health care facilities to initiate action plans and take continuous improvement actions. Obviously, learning-by-doing in a dynamic situation is the essence of the HealthWISE application.

Theoretically, the rapid assessment of the HealthWISE application in China can provide information for health-related action research, which is particularly suited to identifying problems in clinical practice and developing potential solutions to improve practice. Action research is a style of research that typically draws on qualitative methods, such as interviews and observations, rather than by applying a specific method (38).

Our study was carried out with particular attention paid to the following three important elements of action research: (a) the participatory character, which demands that participants perceive the need to change and be willing to play an active part in the research and the change process; (b) a democratic impulse, which usually requires participants to be seen as equals, while the researcher works as a facilitator of change; and (c) the contribution to social science and social change, in that the researcher strives to include the participants' perspectives in the data by feeding back findings to participants (39).

It has been noted that some issues were answered by only one out of ten respondents, in particular, the statements covered suggestions for improving the workshop, because the question was open-ended, a possible explanation for such result might be that, the participants felt satisfaction at the HealthWISE TOT workshop in general, few of them presented their suggestions.

### Monitoring changes in multiple aspects at the hospital

For the purpose of improving the occupational health of HWs, this study showed that significant progress has been made in the Hospital as a whole in the application of HealthWISE, with substantial evidence of its value being available, including the knowledge gathered from the HealthWISE TOT workshop, the actions taken after the HealthWISE TOT workshop, and the comprehensive measures effectively supporting the fight against COVID-19.

Through a mixed qualitative study using interviews and observations, we rapidly assessed changes in the Hospital ranging from those affecting the whole organizational system down to individuals' behaviors and collected opinions containing information of both processes and outcomes. As a result, several exemplary experiences of HealthWISE application in the Hospital can be shared with other pilot hospitals and beyond, which have been summarized as follows (29–37).

#### Leadership commitment and staff participation

Effective organizing, management, and full mobilization are prerequisites to improve the working conditions of HWs (40, 41). Leaders of the Hospital participated in the HealthWISE TOT workshop from the beginning, the Occupational Safety and Health Committee played a leading role covering all departments, the Department of Occupational Health functioned as a coordinator and supervisor, and all staff became self-motivated to access occupational health protection.

#### Technical supporting and facilitating

First, the national program team technically supported the Hospital during the progression from training to implementation *via* the comprehensive HealthWISE approach; second, the HealthWISE TOT workshop participants from the Hospital played the roles of trainers and facilitators for colleagues. This experience echoed our previous study on developing the model of a hospital initiative on systematic occupational health in China (42).

#### Establishing an occupational health and safety management system

In the Hospital, a comprehensive mechanism was developed across multiple aspects of occupational hazards prevention and control, covering regular on-the-job training (pre–post, annual, and thematic training), issuing and updating of internal policies (regulations and standards), cooperation between the Department of Occupational Health and the Department of Infection Prevention and Control, rights safeguarding for occupational health by the Trade Union, and occupational exposure management (notification, response, monitoring, and counseling).

#### Creating an occupational safety and health culture

The preventive culture of occupational safety and health among HWs, together with comprehensive management, can further promote the capacity-building of occupational safety and health (43, 44). Under the core value of “Guard for Lives,” the Hospital adopted the vision of “zero tolerance for occupational hazards,” which was integrated into the culture, thus building a climate of support and care for HWs and their families as well as patients.

#### Enhancing HealthWISE training for motivation of improving

In practice, the trust has been developed between HealthWISE and the Hospital As a result, among the nine trainers of HealthWISE TOT workshop, five had participated once, three had participated twice, and one had participated three times. Subsequently, the trainers of HealthWISE TOT workshop trained the other HWs in the Hospital by lecture and field visit.

In contrast, a global self-administered online survey with health care workers and healthcare delivery stakeholders from 161 countries (including China) suggested that (45), the majority of respondents designated most of the health and safety risk parameters as “not acceptable at all.” Circumstances most reported as such included bullying or psychological harassment in the workplace (54%), physical violence and assaults (54%), exposure to blood, bodily fluids and other infectious materials (52%), inadequate sanitation facilities (52%) and sexual harassment (50%).

### Strengthening the application of HealthWISE in China

With respect to the Hospital, the advantage of HealthWISE application should be accepted to keep the benefits flowing. Staff engagement will address the needs implied by the interviews. Importantly, the occupational health and safety management system for HWs is not effective unless accompanied by a positive health and safety culture in the workplace. It was also suggested by the study on HealthWISE in Africa that successful implementation of HealthWISE requires dedicated local team members who will help to facilitate the process by adapting HealthWISE to the workers' occupational health and safety knowledge and skill levels and the cultures and needs of their hospitals, respectively (25).

The application of HealthWISE nationwide in China is still currently in the development stage, and further promotion is necessary. Under the challenging conditions of the COVID-19 pandemic, three landmark events were launched. The eighth and ninth HealthWISE TOT workshops were conducted in Hubei Province, which was the area of China hardest hit by COVID-19. The eighth workshop was an online training session held from August 1 to September 30, 2020 (46), and the ninth session was a face-to-face training course held in June 2021 (47). The contents were expanded from the previous four modules to six modules with the addition of module 7 (working time and family-friendly measures) and module 8 (selecting, storing, and managing equipment and supplies). A workshop on the rapid assessment of HealthWISE application in China was organized in December 2020 in Beijing (48).

As indicated by the analysis of experiences and lessons of occupational health of HWs in China during the COVID-19 pandemic, by enacting comprehensive measures, China was able to minimize the incidence of COVID-19 cases among HWs (13, 49).

According to the suggestions at the national level from this qualitative study, HealthWISE should be promoted to public health institutions, such as centers for disease prevention and control, centers of maternal and child care, and centers for blood collection and supply. It is suggested that online courses should be developed for a great number of medical colleges where future HWs are being educated; this will allow the mindset of occupational health to be formulated from the very beginning. Additionally, self-protection of occupational health is particularly important for physicians working under a multisite practice policy in China (50).

In terms of the long run, it is important to underscore the remarkable progress of legislation in China. There exist >5 laws relevant to the regulation of occupational hazards for HWs, including the prevention and control of occupational diseases, prevention and treatment of infectious diseases, work safety, mental health, and basic medicare and health promotion, respectively. However, overlaps and gaps continue to persist among those laws (51, 52), so better alignment of practical policies/standards among various law systems with accessible basic occupational health services for HWs is critical, as HealthWISE indicated that “changes will be easier to sustain over the long term if you build them into existing structures and procedures.”

As of 21 February 2022, WHO and ILO jointly published a new guide-*Caring for those who care: Guide for the development and implementation of occupational health and safety programmes for health workers*, for developing and implementing stronger occupational health and safety programs for HWs, as the COVID-19 pandemic continues to exert great pressure on them. WHO and ILO recommend developing and implementing sustainable programs for managing occupational health and safety for HWs at national, sub-national and health facility levels. In this guideline, HealthWISE has been taken as an example of the materials must be derived from validated information accepted by experts at the national level, meanwhile, the practice of HealthWISE application in China during the previous years is introduced alongside this (53, 54).

In harmony with the philosophy of occupational health prevention and control, HealthWISE encourages HWs to apply in a dynamic situation based on the risk assessment. Control measures should be defined under the principle of the hierarchy of occupational hazards controls, i.e., the preferred order of selecting control measures from the most effective to the least effective, as follows. First, it is always best to first try to eliminate the hazard; second, when this is not possible, engineering controls may be used to isolate or remove a hazard from the workplace. Third, administrative controls can be used to implement policies, procedures, and training programs to reduce exposure hazards. Fourth, work practice controls should be used to reduce exposure to occupational hazards through changing work practices. Finally, PPE may be used to establish a physical barrier between the HW and the hazard (5).

### Comparison between global studies relevant to HealthWISE

It would be useful to discuss how this study relates to additional two studies of HealthWISE, the study by Wilcox et al. in three African countries (25), and the other study by Zungu et al. in South Africa (26), even if the framings and methodologies of them differ substantially, the comparison would give readers a more comprehensive picture on international research related to HealthWISE.

First, this study is a qualitative study at a Chinese public hospital with a ≥5-year application of HealthWISE by in-depth interviews and observations, through the eyes of the participants with perspectives of go-forward improvements for the program. The Wilcox et al. study studied the implementation of HealthWISE in seven hospitals in three countries, through a multiple-case study and thematic analysis of data collected primarily from focus group discussions and questionnaires. The Zungu et al. study was a cross-sectional study design applying participatory action research in four provinces of South Africa. A semi-structured questionnaire and a qualitative observational HealthWISE walkthrough risk assessment was carried out to collect data on OSH systems in 45 hospitals to identify factors associated with health worker protection.

Second, the three studies followed the similar approach of empowering health workers and managers to achieve better occupational health. In this study, the directors and staff of the Hospital had motivation to implement HealthWISE, they have taken a series of actions for improving the system, which have been shown in the above description. The Wilcox et al. (25) study examined the enabling factors and barriers to the implementation of HealthWISE. Zungu et al. (26) study focused on risk assessment on OSH systems from perspectives of respondents of Provincial Departments of Health and health workers to conduct the OSH inventory based on HealthWISE tool. In our further study, it is suggested to learn from the studies on the enabling factors/ barriers and risk assessment based on specific occupational hazard, in order to implement HealthWISE among more Chinese hospitals.

Third, both the Wilcox et al. (25) study of HealthWISE and this study found that staffing during implementation was an issue; the recommendation from this study in China was that more staff should be involved in the training of HealthWISE, whereas for Wilcox et al. (25) study it was ensuring that workers and their managers were onside such that those who came to the training were not only willing but able to participate in the implementation. Additionally, this study in China emphasized the importance of a comprehensive mechanism across multiple aspects of occupational hazards prevention and control, while the Zungun et al. (26) study found that health facilities in all four provinces had SARS-CoV-2 plans for the general population but no comprehensive OHS plan for health workers.

Global practice demonstrated that HealthWISE is a useful tool for HWs to identify occupational hazards, plan program of occupational protection, and assess improvement. However, effect assessment is under complex context which needs comprehensive consideration, in particular, multi-source data and evidence. Improvement of occupational health for HWs requires holistic approaches, from the perspective of national policies, resources mobilization is critical with the promulgation by laws and standards; from the perspective of global health, capacity building of HealthWISE implementation should be integrated into international network of occupational health for concert action.

### Strengths and limitations

The validity of the conclusions drawn from qualitative research depends upon a clear understanding of the purpose of the research and, hence, the form of outcome it is intended to create. Qualitative research takes an interpretive, naturalistic approach to its subject matter; qualitative researchers study things by attempting to make sense of, or interpret, phenomena with regard to the meanings that people bring to them (55, 56). An important advantage of an observation study is that it can help to overcome discrepancies between what people say and what they actually do (57).

To the best of our knowledge, this is the first investigation of the HealthWISE application in a Chinese pilot hospital as a whole by qualitative analysis, and the combination of interviews with observations enhances the validity of our conclusions. Rapid assessment is particularly meaningful for protecting HWs during the fight against COVID-19; in the long term, it will contribute to the far-reaching scope of the HealthWISE application. In future, it is noted that the thematic analysis process suggested by Braun and Clarke might be described as a stepwise process, but tends to be more iterative, it also calls for reflexivity in terms of understanding and describing the themes from the perspectives of participants.

Nevertheless, this study has several limitations. Our interview was limited to a purposive sampling of 10 HWs in the Hospital without the consideration of data saturation, our sample was relatively small and was confined to sampling standard, there may have been selection bias, the self-assessment could also have been affected by recall bias, including over- or under-reporting. Due to the limitation of study design, our analysis might not be “deeper” enough for interpreting data and providing their underlying meanings. Furthermore, one size does not fit all, caution is required when the results are generalized. Finally, qualitative and quantitative techniques should be regarded as complementary rather than competitive; therefore, further quantitative research is required to fully understand the progress of HealthWISE. In general, there remains a lot of work to do in occupational health and safety of HWs.

## Conclusion

This study demonstrated the systematic improvement of occupational health for HWs by HealthWISE implementation in the Chinese Hospital. The results of this study fully support the expected scale-up implementation of HealthWISE. This WHO/ILO instrument is a practical tool for health institutions, facilitating the approach of learning-by-doing. Using a qualitative study combining interviews with observations, this investigation undertook thematic analyses from perspectives of learning from the HealthWISE TOT workshop, actions after the HealthWISE TOT workshop, practice during the COVID-19 pandemic, and further suggestions. The findings show that the occupational health of HWs has been improved in the Hospital over 5 years, with remarkable changes seen in the areas of leadership commitment and participation, supporting and facilitating, system establishment, and culture creation. However, considering the gap between the current position and the ultimate goal, there is still a long way to go, and HWs should be guarded against complacency. Recommendations were provided herein for the sustainable application of HealthWISE in China, including maintaining total staff engagement under a positive safety and health culture in the Hospital, promoting HealthWISE to nationwide public health institutions, developing online courses for medical colleges, focusing on alignment between various law systems, and adopting measures under the principle of the hierarchy of occupational hazards controls. The valuable experiences and lessons gathered to date can be shared with other pilot hospitals in China and beyond, especially given the unprecedented challenge of COVID-19. More quantitative research is needed for further understanding of the effectiveness and barriers in order to achieve the goals of safety, health, and well-being for HWs by building a resilient health system.

## Data availability statement

The original contributions presented in the study are included in the article/supplementary material, further inquiries can be directed to the corresponding author/s.

## Ethics statement

The studies involving human participants were reviewed and approved by the Ethical Review by PUMC through approval of the ILO and PUMC program of rapid assessment of the HealthWISE application in China according to internal regulations. All personal identifiers (name, contact information) of participants were removed from the dataset before analysis. Written informed consent for participation was not required for this study in accordance with the national legislation and the institutional requirements.

## Author contributions

MZ was the principal investigator of the study, responsible for and the main contributor to all phases of the study, including the study design, quality assessment, and manuscript modification. YH, FW, DL, CW, and YQ collected the data. YH and MZ analyzed the data and drafted the manuscript. MZ and YH review the thematic analysis. All authors approved the final manuscript for publication.

## Funding

This study was funded by the International Labor Organization and Peking Union Medical College Grant Agreement (No. 2020P216QG014) and the Project of International Expert Consultation for the National Occupational Health System Innovation funded by the Ministry of Science and Technology of the People's Republic of China in 2022–2023 (Grant no. G2022194003L). Funding for article publication fees was provided by the Program of the School of Population Medicine and Public Health, Peking Union Medical College (No. XK-008-ZM).

## Conflict of interest

The authors declare that the research was conducted in the absence of any commercial or financial relationships that could be construed as a potential conflict of interest.

## Publisher's note

All claims expressed in this article are solely those of the authors and do not necessarily represent those of their affiliated organizations, or those of the publisher, the editors and the reviewers. Any product that may be evaluated in this article, or claim that may be made by its manufacturer, is not guaranteed or endorsed by the publisher.
